# Supplementary material for: Association between the Processed Dietary Pattern and Tumor Staging in Patients Newly Diagnosed with Head and Neck Squamous Cell Carcinoma
Source: Cancers (Basel). 2023 Feb 25;15(5):1476. doi: 10.3390/cancers15051476 (PMC10001054; doi:10.3390/cancers15051476)
Supplement: Supplementary file 1 [file cancers-15-01476-s001.zip › cancers-2133321-supplementary.pdf]

Article title: Association Between Processed Dietary Pattern and Tumor Staging in Patients Newly Diagnosed with Head and Neck Squamous Cell Carcinoma

Journal name: Cancers

Author names: Ana Carolina da Silva Lima [1,†], Tathiany Jéssica Ferreira [2,†], Adriana Divina Silva Campos [2], Larissa Morinaga Matida [1], Maria Beatriz Trindade Castro [2], Ana Amélia Freitas-Vilela [3] and Maria Aderuza Horst [1,*]

Affiliation:

[1] Faculty of Nutrition, Federal University of Goiás, 74605-080, Goiânia, Brazil; anacarolinalima@discente.ufg.br (A.C.S.L.); larissamorinaga@discente.ufg.br (L.M.M.); aderuza@ufg.br (M.A.H.)
[2] Josué de Castro Nutrition Institute, Federal University of Rio de Janeiro, 21941-901, Rio de Janeiro, Brazil; tathianyferreira@ufrj.br (T.J.F.); acampos@ufrj.br (A.D.S.C.); mbtcastro@nutricao.ufrj.br (M.B.T.C.)
[3] Special Academic Unit of Health Sciences, Federal University of Goiás, 75801-615, Jataí, Brazil; anaameliafv@ufg.br
* Correspondence: aderuza@ufg.br (M.A.H.); Tel.: +55 62 98250-9000
† These authors contributed equally to this work

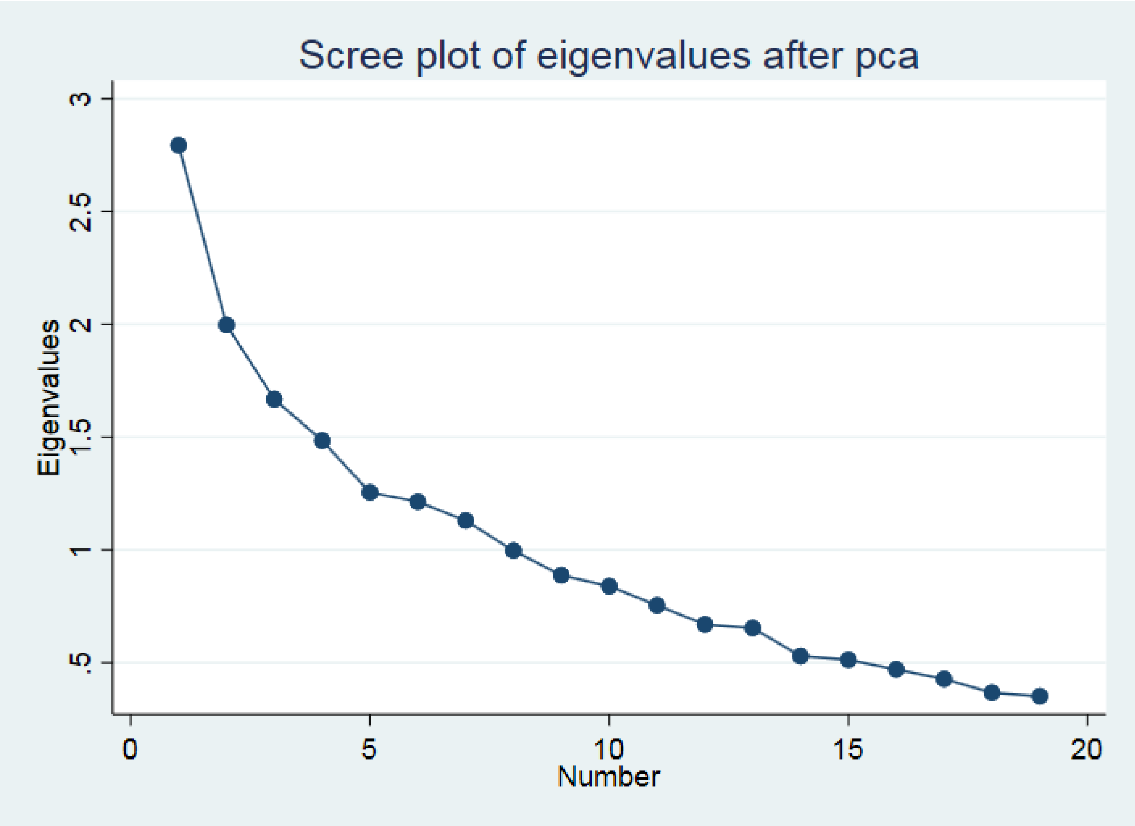

Figure S1. KMO test.

Table S1. Factorial loadings and communalities (h^2) of the three patterns identified in patients with HNSCC.

**Food**	**Factorial loadings and communalities**			
	**Healthy**	**Processed**	**Mixed**	**h^2**
Fruit	**0.469**	-0.081	-0.155	0.483
Tuber	**0.462**	0.063	0.114	0.441
Dairy products	**0.398**	0.156	-0.027	0.522
Fish	**0.316**	-0.160	0.052	0.767
Grains	**0.308**	-0.0612	0.057	0.788
Vegetables and legumes	**0.232**	0.176	0.065	0.753
Fast Food	0.016	**0.418**	-0.186	0.565
Fats	0.046	**0.407**	-0.095	0.608
Breads	0.092	**0.348**	-0.071	0.679
Sugar-sweetened beverages	-0.192	**0.330**	0.063	0.734
Cakes and cookies	0.053	**0.316**	0.051	0.747
Not tender meat	-0.109	**0.302**	0.106	0.778
Tender meat	0.144	**0.202**	0.092	0.808
Beer and wine	-0.003	-0.145	**0.495**	0.484
Distilled beverages	-0.034	-0.074	**0.485**	0.524
Pasta and flour	0.194	0.042	**0.367**	0.618
Bean	-0.100	0.199	**0.367**	0.623
Rice	0.067	0.060	**0.277**	0.816
Coffee	-0.147	0.176	**0.226**	0.801
Number of the groups	6	6	6	

Eigenvalues	2.79	1.99	1.67
% explained variance	11.93	11.50	10.57
% cumulative variance	11.93	23.43	34.00
